# Genetic spectrum of *NOTCH3* and clinical phenotype of CADASIL patients in different populations

**DOI:** 10.1111/cns.13917

**Published:** 2022-07-13

**Authors:** Wang Ni, Yi Zhang, Liang Zhang, Juan‐Juan Xie, Hong‐Fu Li, Zhi‐Ying Wu

**Affiliations:** ^1^ Department of Neurology and Department of Medical Genetics in the Second Affiliated Hospital, and Key Laboratory of Medical Neurobiology of Zhejiang Province Zhejiang University School of Medicine Hangzhou China

**Keywords:** CADASIL, clinical features, genetic spectrum, *NOTCH3*

## Abstract

**Introduction:**

Cerebral autosomal‐dominant arteriopathy with subcortical infarcts and leukoencephalopathy (CADASIL) is a relatively common cerebral small vessel disease. *NOTCH3* has been identified as the causative gene of CADASIL. Clinical variability and genetic heterogeneity were observed in CADASIL patients and need to be further clarified.

**Aims:**

The aim of the study was to clarify genetic spectrum of *NOTCH3* and clinical phenotype of CADASIL patients.

**Methods:**

Suspected CADASIL patients were collected by our center between 2016 and 2021. Whole exome sequencing was performed to screen *NOTCH3* mutations of these patients. Genetic and clinical data of CADASIL patients from previous studies were also analyzed. Studies between 1998 and 2021 that reported more than 9 pedigrees with detailed genetic data or clinical data were included. After excluding patients carrying cysteine‐sparing mutations, genetic data of 855 Asian pedigrees (433 Chinese; 226 Japanese, and 196 Korean) and 546 Caucasian pedigrees, in a total of 1401 CADASIL pedigrees were involved in mapping mutation spectrum. Clinical data of 901 Asian patients (476 Chinese patients, 217 Japanese patients, and 208 Korean patients) and 720 Caucasian patients, in a total of 1621 patients were analyzed and compared between different populations.

**Results:**

Two novel mutations (c.400T>C, p.Cys134Arg; c.1511G>A, p.Cys504Tyr) and 24 known cysteine‐affecting variants were identified in 36 pedigrees. Genetic spectrums of Asians (Chinese, Japanese, and Korean) and Caucasians were clarified, p.R544C and p.R607C were the most common mutations in Asians while p.R1006C and p.R141C in Caucasians. For clinical features, Asians were more likely to develop symptoms of TIA or ischemic stroke (*p* < 0.0001) and cognitive impairment (*p* < 0.0001). Nevertheless, Caucasians had a higher tendency to present migraine (*p* < 0.0001) and psychiatric disturbance (*p* < 0.0001). The involvement of temporal pole was more likely to happen in Caucasians (*p* < 0.0001).

**Conclusion:**

The findings help to better understand the clinical variability and genetic heterogeneity of CADASIL.

## Graphical abstract

Two novel mutations (c.400T>C, p.Cys134Arg; c.1511G>A, p.Cys504Tyr) and 24 known cysteine‐affecting variants were identified in the newly recruited CADASIL patients. The genetic spectrum of NOTCH3 and clinical features of CADASIL patients were analyzed and summarized in different populations. The study help to understand the clinical variability and genetic heterogeneity of CADASIL.

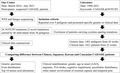

## 1 INTRODUCTION

Cerebral autosomal‐dominant arteriopathy with subcortical infarcts and leukoencephalopathy (CADASIL) is an autosomal‐dominant hereditary disease of cerebral small vessels. It is the most common heritable disease that causes stroke and vascular dementia. *NOTCH3* has 33 exons and encodes a single‐pass transmembrane Notch3 receptor. It has been identified as the causative gene of CADASIL.^1^ The 2–24 exons of *NOTCH3* encode 34 epidermal growth factor‐like repeats (EGFR) in extracellular domain.^2^ Each EGFR contains 6 cysteine residues which form 3 disulfide bonds, stabilizing the Notch3 receptor. Mutations resulting in cysteine number alteration from even to odd within EGFR change the structure of the extracellular domain of Notch3 receptor and lead to misfolding and aggregation of the extracellular domain. This may cause granular osmiophilic material (GOM) deposits which is a hallmark of CADASIL and degrade vascular smooth muscle cells.^3^, ^4^ Gene test for *NOTCH3* is the gold standard for CADASIL diagnosis.

Typical symptoms of CADASIL patients including migraine with aura, transient ischemic attacks (TIA) or ischemic stroke, intracranial hemorrhage, cognitive impairment, and psychiatric disturbance. Patients may present one or more symptoms above. Brain MRI of CADASIL patients are characterized with white matter hyperintensities, subcortical infarcts, and cerebral microbleeds.^5^ Abnormalities in periventricular areas are early features in T2‐weighted imaging and fluid‐attenuated inversion recovery. These abnormalities later diffuse symmetrically to other areas including characteristic locations of external capsule and anterior temporal pole.^6^

Previously, we have reported a multicenter cohort of 214 Chinese patients with CADASIL collected from 2009 to 2016,^7^ which broadened the mutation spectrum of *NOTCH3* and further confirmed the founder effect of p.Arg544Cys in Chinese patients. However, the preceding works did not clarify the clinical difference between Asians and Caucasians. Herein, we collected genetic and clinical data of CADASIL patients from our center during the past 6 years and summarized data from large cohorts reported previously. Genetic spectrums of CADASIL patients in different populations were mapped, and difference between populations was highlighted.

## 2 MATERIALS AND METHODS

### 2.1 Study subjects

According to diagnostic criteria for CADASIL,^8^ clinically suspected CADASIL patients were consecutively collected from the Second Affiliated Hospital between March 7, 2016, and July 2, 2021. After excluding patients carrying pathogenic variants of causative genes for familial Alzheimer's disease and hereditary leukodystrophies,^9^, ^10^ 36 pedigrees were genetically diagnosed and recruited in this study. These patients were primarily from Zhejiang Province (~80%). Clinical and genetic data were summarized and analyzed. The study was approved by the ethics committee of the Second Affiliated Hospital of Zhejiang University School of Medicine. Each participant signed a written informed consent, respectively.

### 2.2 Genetic analysis

Genomic DNA from patients was extracted from peripheral blood using QIAamp DNA Blood Minikit (QIAGEN, Hilden, Germany). Whole exome sequencing (WES) was performed on the Illumina HiSeq X Ten platform (XY Biotechnology Co. Ltd.) using Agilent SureSelect™ Human All Exon v6 kit and variants were annotated by ANNOVAR according to the detailed protocol reported previously,^10^, ^11^ potential variants of *NOTCH3* were confirmed by Sanger sequencing using a procedure as previously described^12^ on ABI 3500XL DX DNA sequences (Applied Biosystems). Then, the sequence was compared with human *NOTCH3* sequence on Ensembl (http://asia.ensembl.org). Variants were identified on the Human Gene Mutation Database (HGMD, available at http://www.hgmd.cf.ac.uk/ac/index.php). The pathogenicity of novel variants was predicted through sorting intolerant from tolerant (SIFT), polymorphism phenotyping v2 (Polyphen‐2), Mutation Taster, rare exome variant ensemble learner (REVEL), and MetaLR. The population frequencies of variants were obtained from 1000 Genomes Project (1000 g), Exome Aggregation Consortium (ExAC), and the Genome Aggregation Database (gnomAD). Classification of variants was based on the American College of Medical Genetics and Genomics (ACMG) standards and guidelines.^13^

### 2.3 Data sources

**FIGURE 1:**
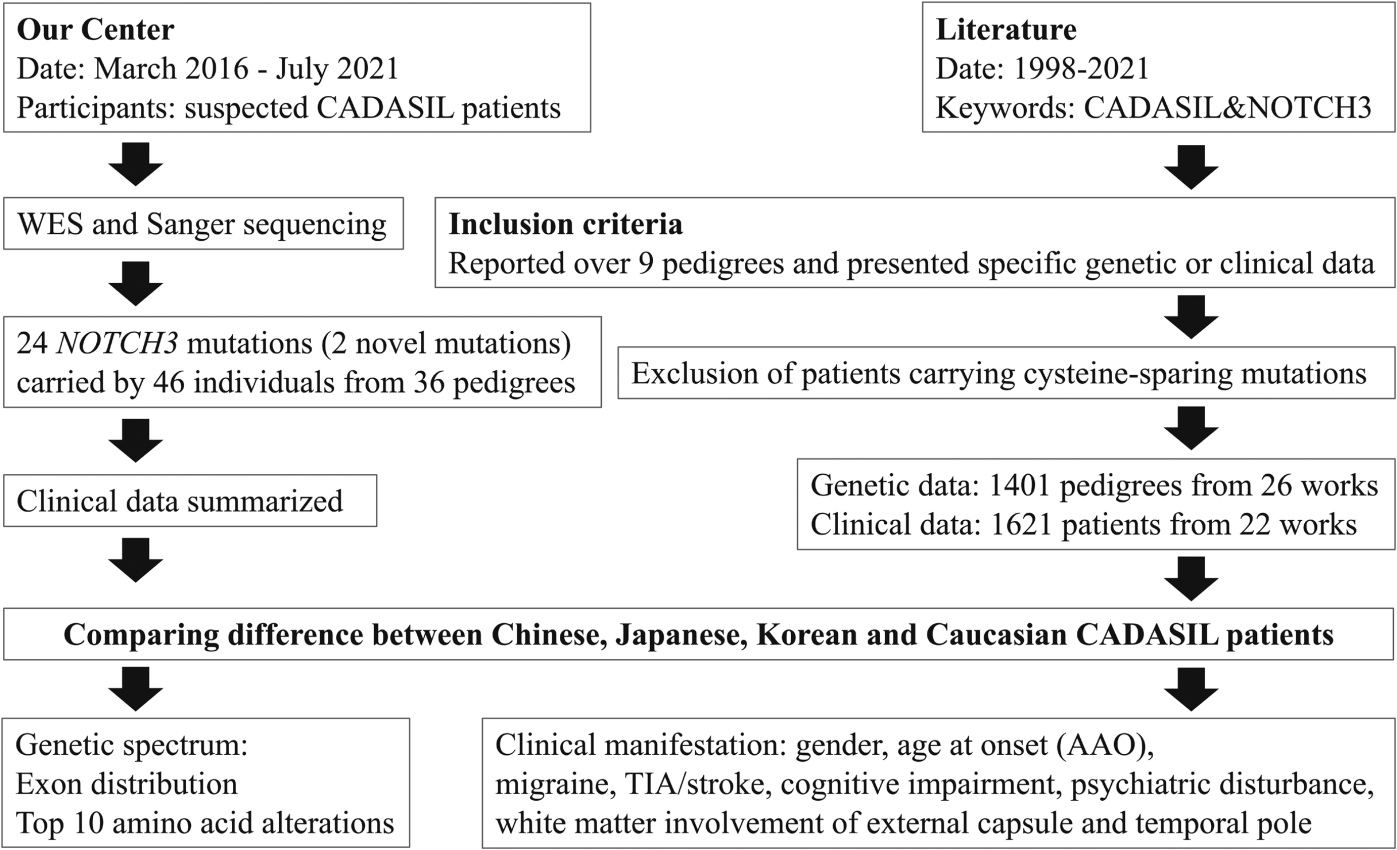
Flowchart of the study design

Data of patients with CADASIL were collected from published articles that reported more than 9 pedigrees, as well as this study. These researches were from Asia and Europe, published from 1998 to 2021. Among all published articles and this study, 26 articles and 22 articles provided detailed genetic data and clinical data, respectively. Despite some works have reported some cysteine‐sparing mutations and mutations outside EGFR, the pathogenicity of these kinds of mutations is still ambiguous, and therefore patients carrying these kinds of mutations were excluded. Genetic data of 1401 pedigrees were involved in mapping mutation spectrum. Clinical data of 1621 patients were analyzed and compared between different populations. The detailed study design is shown in Figure 1.

### 2.4 Statistical analysis

Statistical analyses were performed and graphed using SPSS version 26.0 (SPSS Inc.) and GraphPad Prism 9.3.1 (GraphPad Software). The data normality of age at onset was assessed by the D'Agostino‐Pearson test and was compared by Kruskal–Wallis test. For articles that did not provide specific age of onset for each patient, mean values were used instead. Other clinical manifestations were compared by chi‐square test for categorical variables and adjusted by Bonferroni correction. Values of two‐tailed *p* < 0.05 were considered statistically significant.

## 3 RESULTS

### 3.1 Genetic and clinical features of patients with CADASIL

In the present study, 39 patients and 7 asymptomatic individuals from 36 pedigrees were genetically diagnosed. A total of 24 variants including 22 known variants and two novel ones, c.400T>C (p.Cys134Arg) and c.1511G>A (p.Cys504Tyr) (Table 1), were identified. The two novel variants were absent from 1000 g, ExAC, and gnomAD. According to the ACMG standards and guidelines,^13^ both variants can be defined as “likely pathogenic.” As shown in Table 2, mutations mainly distributed on exon 11 and exon 4, with a proportion of 42.11% and 23.68%, respectively. Among all mutations, c.1630C>T (p.Arg544Cys) had the highest frequency (18.42%) with c.1819C>T (p.Arg607Cys) following behind (15.79%). Both mutations were mapped to exon 11.

**TABLE 1:** Two novel mutations of *NOTCH3* in CADASIL patients

Gender	AAO	Manifestations	Family history	Nucleotide alteration	Amino acid alteration	In silico prediction					Frequency of population			ACMG
						SIFT	Polyphen‐2	Mutation Taster	REVEL	MetaLR	1000 g	ExAC	gnomeAD	
M	48	headache, dizziness, migraine	Yes	c.400T>C	p.C134R	D	PD	D	D	D	—	—	—	LP
M	54	headache, dizziness, ischemic stroke	Yes	c.1511G>A	p.C504Y	D	PD	D	D	D	—	—	—	LP

Abbreviations: 1000 g, 1000 Genomes Project; AAO, age at onset; D, damaging or disease‐causing; ExAC, Exome Aggregation Consortium; gnomAD, Genome Aggregation Database; LP, likely pathogenic; PD, probably damaging; Polyphen‐2, polymorphism phenotyping v2; REVEL, rare exome variant ensemble learner; SIFT, sorting tolerant from intolerant.

**TABLE 2:** Mutation spectrum of CADASIL patients in this cohort

Exon	Nucleotide alteration	Amino acid alteration	EGFR	Pedigrees	Proportion of mutation	Proportion of exon
3	c.268C>T	p.R90C	2	1	2.63%	7.89%
	c.328C>T	p.R110C	2	2	5.26%	
4	c.397C>T	p.R133C	3	2	5.26%	23.68%
	c.400T>C	p.C134R	3	1	2.63%	
	c.430T>A	p.C144S	3	1	2.63%	
	c.457C>T	p.R153C	3	1	2.63%	
	c.505C>T	p.R169C	4	1	2.63%	
	c.544C>T	p.R182C	4	2	5.26%	
	c.602G>C	p.C201S	5	1	2.63%	
6	c.994C>T	p.R332C	8	1	2.63%	2.63%
8	c.1261C>T	p.R421C	10	1	2.63%	2.63%
10	c.1511G>A	p.C504Y	12	1	2.63%	2.63%
11	c.1630C>T	p.R544C	14	7	18.42%	42.11%
	c.1759C>T	p.R587C	15	1	2.63%	
	c.1774C>T	p.R592C	15	1	2.63%	
	c.1817G>T	p.C606F	15	1	2.63%	
	c.1819C>T	p.R607C	15	6	15.79%	
12	c.1918C>T	p.R640C	16	1	2.63%	2.63%
14	c.2149C>T	p.R717C	18	1	2.63%	5.26%
	c.2182C>T	p.R728C	18	1	2.63%	
15	c.2353C>T	p.R785C	20	1	2.63%	2.63%
19	c.3016C>T	p.R1006C	26	1	2.63%	2.63%
20	c.3226C>T	p.R1076C	27	1	2.63%	2.63%
21	c.3427C>T	p.R1143C	29	1	2.63%	2.63%

Abbreviation: EGFR, epidermal growth factor repeats.

Clinical features of 39 symptomatic patients from 36 pedigrees were summarized (Table 3). The average age at onset was 49.77 ± 3.60 while the average age at diagnosis was 53.13 ± 3.35. Among 36 probands, 25 had a family history. Initial symptoms of the 39 patients were collected, 15 (38.46%) complained memory loss, 10 (25.64%), and 2 (5.13%) experienced ischemic stroke and intracranial hemorrhage separately, 6 (15.38%) presented dizziness and 5 (12.82%) had headache. During the disease course, 29 (74.36%) patients had TIA or ischemic stroke at least once, 27 (69.23%) showed cognitive impairment, 19 (48.72%) presented dizziness, 10 (25.64%) showed psychiatric disturbance, and 6 (15.38%) of 10 (25.64%) patients who had headache met the diagnostic criteria of migraine, respectively.

**TABLE 3:** Clinical manifestations of CADASIL patients in this cohort

Clinical features	Total (*n* = 39)	Exon4 (*n* = 9)	Exon11 (*n* = 14)	Other exons (*n* = 14)
Gender (Male/Female)	19/20	5/4	5/9	9/5
Age at onset	49.77 ± 3.60	45.56 ± 8.83	55.43 ± 56.13	46.29 ± 5.64
Age at diagnosis	53.13 ± 3.35	50.22 ± 5.99	56.71 ± 5.70	50.86 ± 6.83
Family history	25/36 (69.44%)	7/9 (77.8%)	10/14 (71.43)	8/11 (72.73%)
Symptoms				
TIA or ischemic stroke	29/39 (74.36%)	8/9 (88.89%)	10/14 (71.43%)	9/14 (64.29%)
Intracranial hemorrhage	2/39 (5.13%)	0	1/14 (7.14%)	1/14 (7.14%)
Headache	10/39 (25.64%)	3/9 (33.33%)	3/14 (21.43%)	3/14 (21.43%)
Migraine	6/39 (15.38%)	2/9 (22.22%)	3/14 (21.43%)	1/14 (7.14%)
Dizziness	19/39 (48.72%)	4/9 (44.44%)	7/14 (50%)	7/14 (50%)
Cognitive impairment	27/39 (69.23%)	6/9 (66.67%)	9/14 (64.29%)	10/14 (71.43%)
Psychiatric disturbance	10/39 (25.64%)	0	6/14 (42.86%)	2/14 (14.29%)
MRI				
WHM involvement	**Total (*n* = 17)**	**Exon4 (*n* = 7)**	**Exon11 (*n* = 5)**	**Other exons (*n* = 3)**
External capsule	14/17 (82.35%)	6/7 (85.71%)	4/5 (80%)	2/3 (66.7%)
Temporal pole	12/17 (70.59%)	5/7 (71.43%)	3/5 (60%)	2/3 (66.7%)
Periventricular area	17/17 (100%)	7/7 (100%)	5/5 (100%)	3/3 (100%)
Frontal lobe	14/17 (82.35%)	7/7 (100%)	4/5 (80%)	2/3 (66.7%)
Brain stem	5/17 (29.41%)	3/7 (42.86%)	0	1/3 (33.3%)
Thalamus	8/17 (47.06%)	3/7 (42.86%)	3/5 (60%)	2/3 (66.7%)
Corpus callosum	6/17 (35.29%)	2/7 (28.57%)	2/5 (40%)	2/3 (66.7%)
Subcortical infarcts	16/17 (94.12%)	7/7 (100%)	4/5 (80%)	3/3 (100%)
Cerebral microbleeds	5/17 (29.41%)	2/7 (28.57%)	3/5 (60%)	0

Brain MRI data were available for 17 patients. 14 (82.35%) and 12 (70.59%) patients showed white matter hyperintensities (WMHs) in external capsule and temporal pole, respectively. WMHs were also observed in frontal lobe of 14 patients (82.35%), brain stem of 5 patients (29.41%), thalamus of 8 patients (47.06%), and corpus callosum of 6 patients (35.29%). Additionally, 16 (94.12%) patients presented multiple small infarcts while 5 (29.41%) presented cerebral microbleeds.

### 3.2 Genetic spectrum of *NOTCH3* in different populations

**FIGURE 2:**
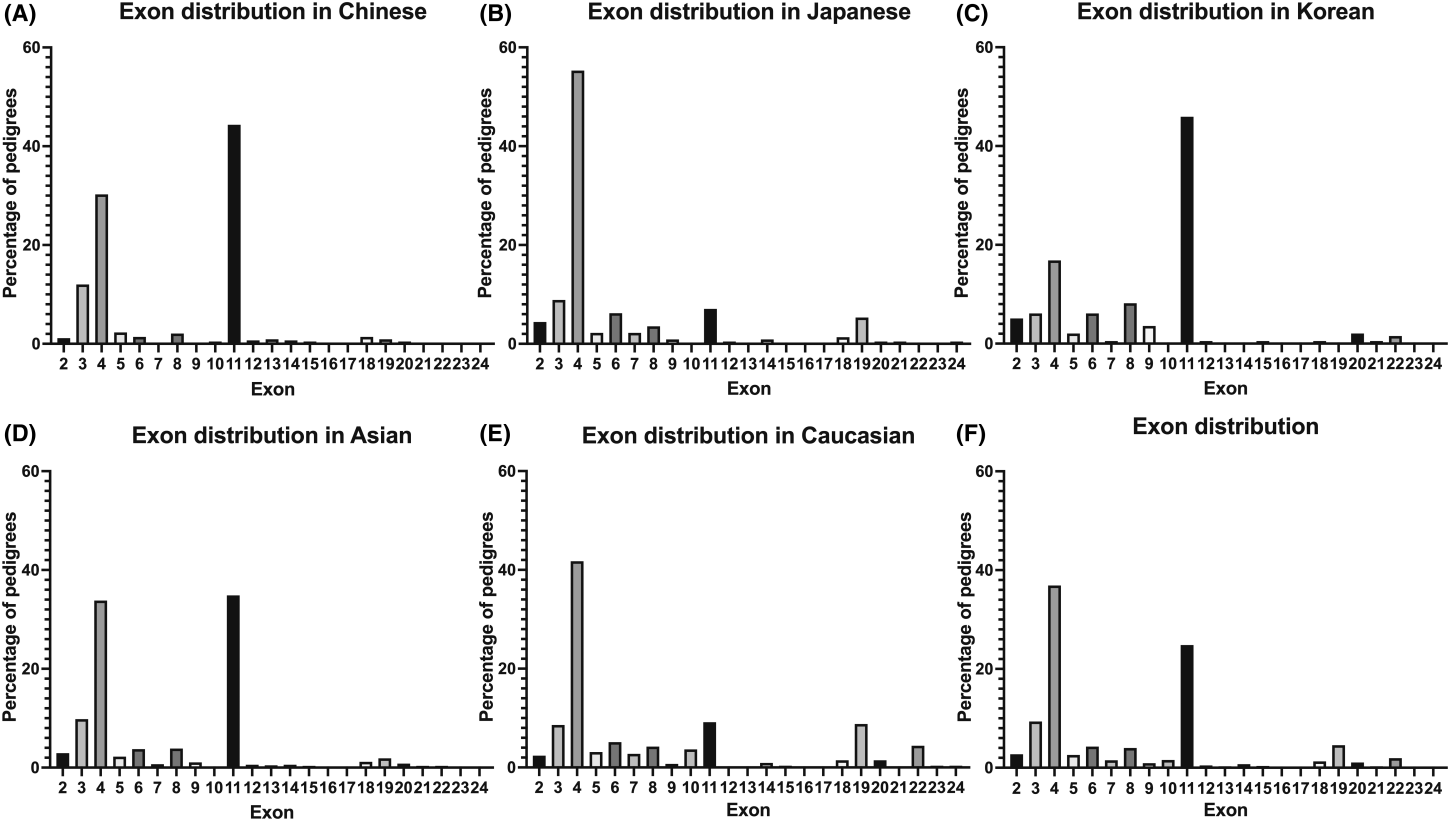
Exon distribution of *NOTCH3* mutations in different populations. (A) Exon distribution of *NOTCH3* mutations in Chinese. (B) Exon distribution of *NOTCH3* mutations in Japanese. (C) Exon distribution of *NOTCH3* mutations in Korean. (D) Exon distribution of *NOTCH3* mutations in Asian. (E) Exon distribution of *NOTCH3* mutations in Caucasian. (F) Exon distribution of *NOTCH3* mutations in all populations

Additionally, we collected the genetic data of 1401 CADASIL pedigrees in different populations, including 433 Chinese, 226 Japanese, 196 Korean, and 546 Caucasian pedigrees, previously reported (Table S1). A total of 247 variants were identified. Among 91 variants from 433 Chinese pedigrees, mutations on exon 11 occupied a larger proportion (44.34%) than exon 4 (30.25%) and exon 3 (12.01%) (Figure 2A). More specifically, mutations of patients from Southern China were more distributed on exon 11 (49.44%) than exon 4 (26.69%) and exon 3 (10.96%), which was different from Northern China (exon 3, 16.88%; exon 4, 46.75%; exon 11, 20.78%). Among 76 variants from 226 Japanese pedigrees, 55.31% of Japanese patients carried mutations on exon 4, and mutations on exon 3 and exon 11 only accounted for 8.85% and 7.08% (Figure 2B). Among 50 variants from 196 Korean pedigrees, 45.92% of mutations were on exon 11 and 16.84% were on exon 4 (Figure 2C). To sum up, 165 variants were found in 855 Asian pedigrees, 33.80% located on exon 4 and 34.85% located on exon 11, and 9.82% on exon 3 (Figure 2D). The analysis was extended to 546 Caucasian pedigrees, in whom 137 variants were found, 41.76% located on exon 4, with exon 11 (9.16%), exon 19 (8.79%), and exon 3 (8.61%) following behind (Figure 2E). As shown in Figure 2F, exon 3, exon 4, and exon 11 were mutation hotspots with a percentage of 9.35%, 36.90%, and 24.84% in several in the world. While Chinese especially Southern Chinese and Korean population had a higher frequency of mutations on exon 11, most of the mutations located on exon 4 in Northern Chinese, Japanese, and Caucasian populations.

**FIGURE 3:**
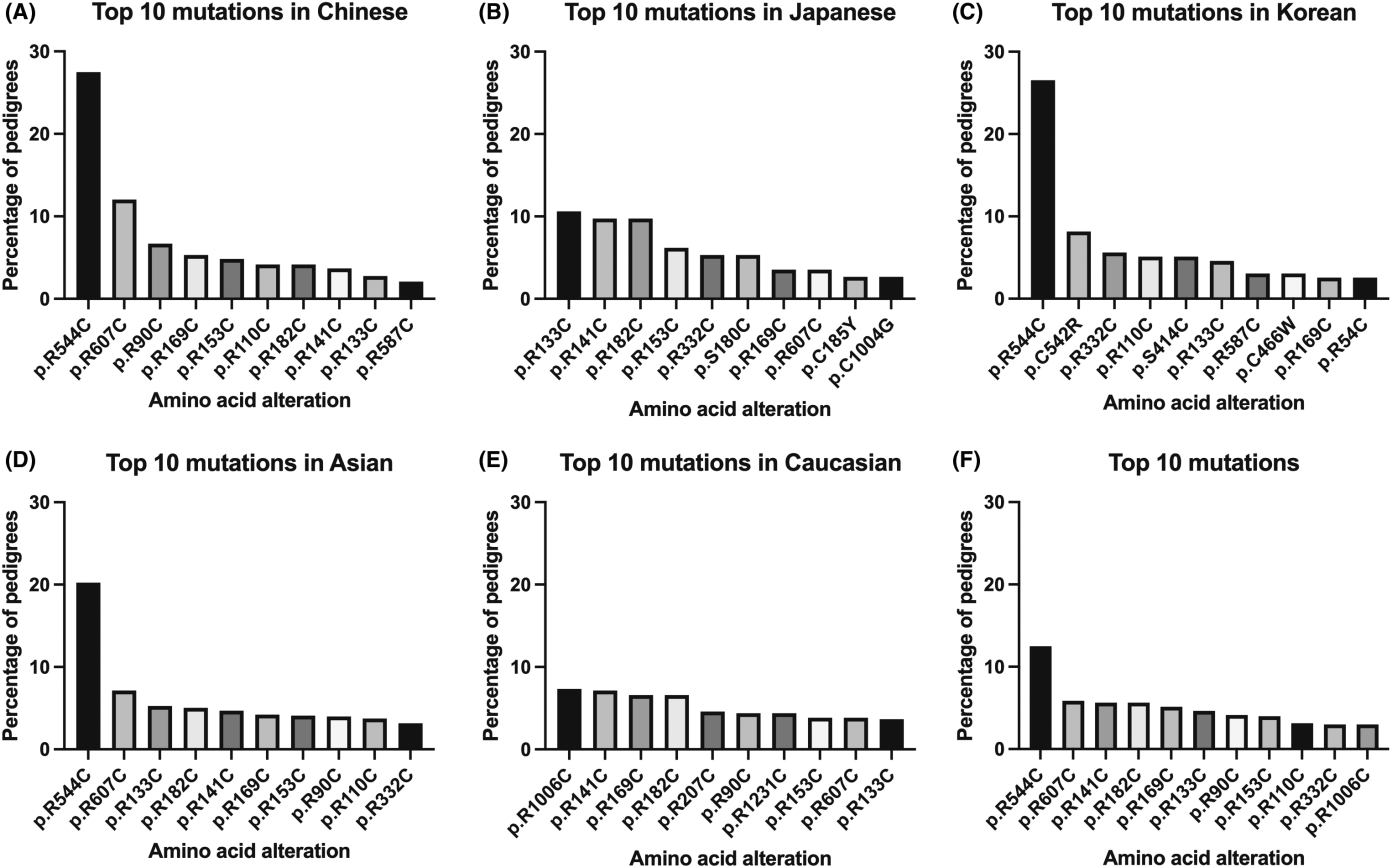
Top 10 mutations in different populations. (A) Top 10 mutations in Chinese. (B) Top 10 mutations in Japanese. (C) Top 10 mutations in Korean. (D) Top 10 mutations in Asian. (E) Top 10 mutations in Caucasian. (F) Top 10 mutations in all populations

**FIGURE 4:**
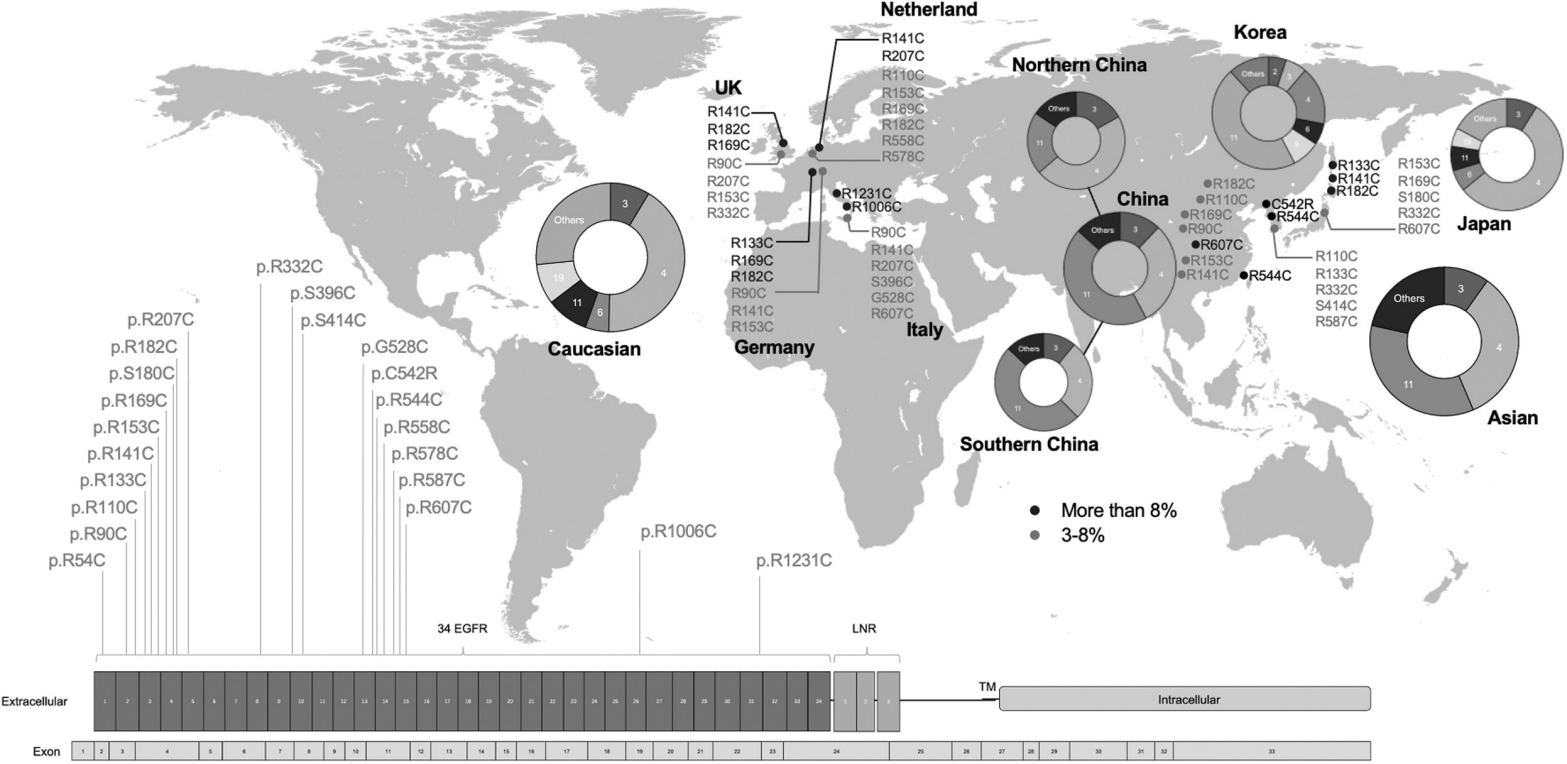
Geographical genetic map of *NOTCH3* mutations. Bottom: schematic representation of the *NOTCH3* with mutations reported over 10 cases worldwide. Top: The proportion of a specific mutation to all mutations in each country was calculated and the mutation was colored in black (>8%) or gray (3–8%) according to the proportion. The location of the mutation on the map reflects only the country in which it was reported and not the exact location within the country. Exon distribution was shown in the form of doughnut chart

The top 10 mutations of different populations were presented in Figure 3. In China, p.Arg544Cys (27.48%) and p.Arg607Cys (12.01%) were the most two common mutations (Figure 3A). Patients carrying p.Arg544Cys accounted for large proportion of all patients in Southern China (32.58%) especially Taiwan (70.53%), while the proportion in Northern China was only 3.90%. p.Arg607Cys was prevalent in both Southern and Northern China (11.80% and 12.99%), as well as p.R90C (6.46% and 7.79%), p.R169C (5.06% and 6.49%), p.R153C (4.77% and 5.19%), p.R110C (3.93% and 5.19%), and p.R141C (3.65% and 3.90%). Besides, p.R182C (3.09% and 9.09%) was more prevalent in the North while patients carrying p.R133C (3.09% and 1.30%) and p.R587C (2.25% and 1.30%) were more from the South. In Japan, the most common mutations were p.Arg133Cys (10.62%), p.Arg141Cys (9.73%), and p.Arg182Cys (9.73%), all of them located on exon 4 **(**Figure 3B). Interestingly, p.Ser180Cys and p.Cys1004Gly were two specific mutations in Japan. In Korea, the proportion of p.Arg544Cys (26.53%) was much higher than other mutations (Figure 3C), in which p.Cys542Arg, p.Ser414Cys, and p.Cys466Trp were unique mutations. The top 10 mutations of Asian, Caucasian, and all populations were summarized in Figure 3D, Figure 3E, and Figure 3F, respectively. p.Arg544Cys (20.23%) and p.R607C (7.13%) in exon 11 were the most common mutations in Asia. Regarding the Caucasian population, the most common mutations were p.Arg1006Cys (7.33%) and p.Arg141Cys (7.24%) located in exons 19 and 4, respectively. But the following two mutations, p.Arg169Cys (6.59%), p.Arg182Cys (6.59%) were also common in in Caucasians. On the whole, spectrum of Asian and Caucasian shared some similarities. Such as, p.Arg607Cys, p.Arg182Cys, p.Arg90Cys, p.Arg169Cys, p.Arg153Cys, p.Arg141Cys, and p.Arg133Cys were all arginine‐cysteine transition and among top 10 mutations in both Asian and Caucasian populations. However, p.Arg544Cys, p.Arg110Cys, and p.Arg332Cys were less common in Caucasian populations while p.Arg1006Cys, p.Arg207Cys and p.Arg1231Cys were less common in Asian populations. A geographical genetic map was shown in Figure 4. Mutations reported more than 10 cases worldwide were mapped. The proportion of a specific mutation to all mutations in each country was calculated and was colored in black (>8%) or gray (3%–8%). Exon distribution in the form of doughnut chart was also shown.

### 3.3 Clinical features of CADASIL patients in different populations

**FIGURE 5:**
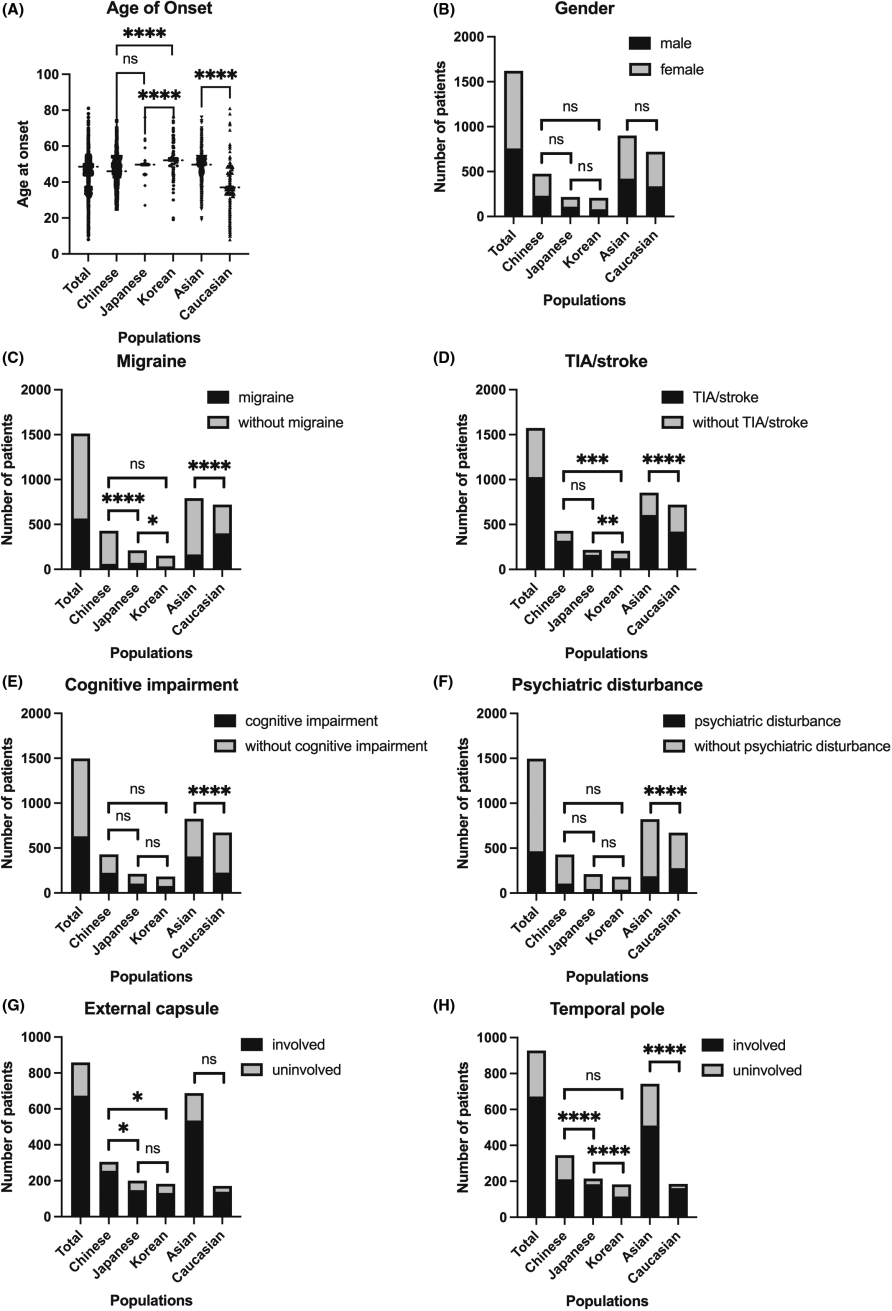
Clinical features of CADASIL patients in different populations. (A) Age at onset. (B) Gender. (C) Number of patients with the symptom of migraine. (D) Number of patients with the symptom of TIA/stroke. (E) Number of patients with the symptom of cognitive impairment. (F) Number of patients with the symptom of psychiatric disturbance. (G) Number of patients with white matter involvement in external capsule. (H) Number of patients with white matter involvement in temporal pole. ns: not significant; *p < 0.05; **p < 0.01; **p < 0.001; ****p < 0.0001

Clinical features of 1621 patients reported previously were summarized, including gender, age at onset (AAO), family history, migraine, TIA/stroke, cognitive impairment, psychiatric disturbance, white matter involvement of external capsule and temporal pole (Table 4). 476 Chinese patients, 217 Japanese patients, 208 Korean patients, and 720 Caucasian patients were included in the study. Detailed statistics were presented in Table S2. We firstly compared the clinical manifestations between patients from Southern and Northern China. There was no significant difference in gender (48.75% vs. 43.86%), incidence of migraine (14.54% vs. 14.91%), and white matter involvement of external capsule (84.47% vs. 88.68%) and temporal pole (72.94% vs. 78.71%). Though slight difference was detected in the incidence of TIA/stroke (73.21% vs. 82.46%) (*p* = 0.0484), cognitive impairment (52.30% vs. 63.16%) (*p* = 0.0427), and psychiatric disturbance (25.77% vs. 8.82%) (*p* = 0.0017). Next, we found that Asians (Chinese 46.23 ± 7.54; Japanese 48.74 ± 2.41; Korean 52.23 ± 0.48) had a later AAO compared with Caucasians (39.15 ± 6.62) (*p* < 0.0001). Korean patients were the latest to develop the disease among the three Asian countries (Chinese vs. Korean, *p* < 0.0001; Japanese V.S. Korean, *p* < 0.0001) (Figure 5A). There was no significant difference in the sex ratio between populations (Figure 5B). For family history, a high proportion of Caucasian had a positive one (96.32%) which was significantly different from Asian (61.92%) (*p* < 0.0001). Japanese patients (87.27%) were closer to Caucasians rather than Chinese (54.5%) and Korean (46.59%) (Chinese vs. Japanese, *p* < 0.0001; Japanese vs. Korean *p* < 0.0001). Regarding the clinical symptoms presentation, chi‐square tests also indicated that Asians had a lower tendency to present migraine than Caucasians (20.71% vs. 55.39%) (*p* < 0.0001). The proportion of patients with migraine was lowest in China (13.99%), followed by Korea (21.57%) and Japan (34.12%), with significant differences between Japan and the other two countries (Chinese vs. Japanese, *p* < 0.0001; Japanese vs. Korean, *p* = 0.01) (Figure 5C). Nevertheless, Asians were more likely to develop symptoms of TIA or ischemic stroke than Caucasians (70.96% vs. 58.47%) (*p* < 0.0001). But Korean patients showed a lower rate of TIA or ischemic stroke (59.62%) compared with Chinese (74.36%) and Japanese (75.12%) (Chinese vs. Korean, *p* = 0.0002; Japanese vs. Korean, *p* = 0.0009) (Figure 5D). While Asian patients were more likely to develop cognitive impairment (49.09% vs. 33.48%) (*p* < 0.0001) (Figure 5E), Caucasian patients had psychiatric disturbance more (22.84% vs. 41.22%) (*p* < 0.0001) (Figure 5F). Chinese patients had the highest percentage of cognitive impairment (52.21%), followed by Japanese (48.83%) and Korean (42.08%), but there was no significant difference. The proportion of psychiatric disturbance in Asian countries were all near 20% and did not have significant difference between each other. For MRI results, there was no significant difference of external capsule involvement between Asians and Caucasians (77.76% vs. 81.29%) (*p* = 0.3503). But Chinese seemed to have a higher possibility of external capsule involvement (83.61%) compared with Japanese (74%) and Korean (72.13%) (Chinese vs. Japanese, *p* = 0.0093; Chinese vs. Korean, *p* = 0.0038) (Figure 5G). The involvement of temporal pole was different between Asians and Caucasians (68.64% vs. 87.57%) (*p* < 0.0001). Japanese patients had a higher incidence of temporal pole involvement (85.58%) than Chinese (61.16%) and Korean (62.84%) (Chinese vs. Japanese, *p* < 0.0001; Japanese vs. Korean, *p* < 0.0001) and was closer to Caucasian (Figure 5H).

**TABLE 4:** Clinical manifestations of different populations

Population	Total	Chinese	Japanese	Korean	Asian	Caucasian	*p* value (Chinese vs. Japanese)	*p* value (Chinese vs. Korean)	*p* value (Japanese vs. Korean)	*p* value (Asian vs. Caucasian)
Patients	1621	476	217	208	901	720				
Gender	757/1621 (46.7%)	231/476 (48.53%)	109/217 (50.23%)	81/208 (38.94%)	421/901 (46.73%)	336/720 (46.67%)	0.6830	0.0242	0.0247	>0.9999
Age at onset	44.19 ± 7.78	46.23 ± 7.54	48.74 ± 2.41	52.23 ± 0.48	48.22 ± 6.11	39.15 ± 6.62	0.9102	**<0.0001**	**<0.0001**	**<0.0001**
Family history	466/677 (68.83%)	109/200 (54.5%)	144/165 (87.27%)	82/176 (46.59%)	335/541 (61.92%)	131/136 (96.32%)	**<0.0001**	0.1478	**<0.0001**	**<0.0001**
Clinical symptom										
Migraine	565/1513 (37.34%)	60/429 (13.99%)	72/211 (34.12%)	33/153 (21.57%)	165/793 (20.81%)	400/720 (55.56%)	**<0.0001**	0.0391	**0.0100**	**<0.0001**
TIA/Ischemic stroke	1027/1574 (65.25%)	319/429 (74.36%)	163/217 (75.12%)	124/208 (59.62%)	606/854 (70.96%)	421/720 (58.47%)	0.8489	**0.0002**	**0.0009**	**<0.0001**
Cognitive impairment	630/1497 (42.08%)	224/429 (52.21%)	104/213 (48.83%)	77/183 (42.08%)	405/825 (49.09%)	225/672 (33.48%)	0.4507	0.022	0.1895	**<0.0001**
Psychiatric disturbance	465/1495 (31.1%)	105/429 (24.48%)	46/211 (21.8%)	37/183 (20.22%)	188/823 (22.84%)	277/672 (41.22%)	0.4895	0.2957	0.7122	**<0.0001**
WMH involvement										
External capsule	674/859 (78.46%)	255/305 (83.61%)	148/200 (74%)	132/183 (72.13%)	535/688 (77.76%)	139/171 (81.29%)	**0.0093**	**0.0038**	0.7297	0.3503
Temporal pole	672/928 (72.41%)	211/345 (61.16%)	184/215 (85.58%)	115/183 (62.84%)	510/743 (68.64%)	162/185 (87.57%)	**<0.0001**	0.7778	**<0.0001**	**<0.0001**

Abbreviation: WMH, white matter hyperintensity.

## 4 DISCUSSION

CADASIL is the most common hereditary disease of cerebral small vessels. Our center has been collecting data of CADASIL patients since 2009 and previously, we published the clinical and genetic data of the CADASIL patients recruited until 2016.^7^ In the present study, we updated our CADASIL patient's cohort by summarizing the clinical and genetic data of the CADASIL patients recruited between 2016 and 2021. In addition, we also summarized the genetic and clinical data from previous studies, involving relatively large cohorts of patients from different populations. This study provides a clearer genetic spectrum and clinical features of CADASIL patients with a particular focus on Asia and Caucasian populations.

There has been some previous work comparing differences between cohorts but not between large populations. Our study suggested that the genetic spectrum of CADASIL patients was different both between Asians and Caucasians and within Asians. Exon 11 and exon 4 is the hotspot of Korea and Japan, respectively, but are both of Chinese. Exon 4 is the classical mutation hotspot of Caucasians. The p.Arg607Cys, p.Arg182Cys, p.Arg90Cys, p.Arg169Cys, p.Arg153Cys, p.Arg141Cys, and p.Arg133Cys, are among top 10 mutations in both Asians and Caucasians. However, the frequency of p.Arg544Cys, p.Arg110Cys, p.Arg332Cys, p.Arg1006Cys, p.Arg207Cys, and p.Arg1231Cys are quite different between Asians and Caucasians. The existence of founder effect of p.Arg544Cys and p.Arg1006Cys was discussed in previous work.^7^, ^14^ Differences also exist in clinical manifestations among the populations. The significant difference between family history may related to relatively small sample size of each population. Future studies are needed to confirm the relationship between family history and populations. Asian patients especially Koreans had a later AAO. It is of strong possibility that the later AAO of Koreans is associated with mutations of p.Arg544Cys.^14^, ^15^ Previous work has pointed out that the proportion of migraine with aura was 20%–40% in Caucasian while in Chinese patients it was often around or lower than 20%.^7^, ^16^, ^17^, ^18^, ^19^ Our results showed that the proportion in Caucasian was over 50%, which meant the difference between Asians and Caucasians might bigger than we know. Also, work from Taiwan pointed out that less frequent temporal pole involvement was associated with p.Arg544Cys.^14^ Our result also showed that temporal pole white matter was less possibly to be involved in Chinese and Korean, the two populations with high frequency of p.Arg544Cys, which is in correspondence to previous works.^14^ Apart from that, our work has shown that the occurrence rates of other typical symptoms are also different between populations. For Chinese and Japanese patients, TIA/stroke are more likely to occur. Cognitive impairment and psychiatric disturbance are of higher possibilities in Asians and Caucasians, respectively. Recent work also suggests that Asian people have a lower prevalence of psychiatric symptom.^19^ This provides more references for CADASIL diagnosis.

Recent studies have discussed more about the role of cysteine‐sparing mutations of *NOTCH3* in CADASIL pathogenesis since patients carrying those mutations presented typical CADASIL symptoms.^20^, ^21^ Some mutations seem to be disease‐causing with evidence come to light. For example, p.Arg75Pro, a common mutation in Asia, especially Japan and Korea, was proven to be associated with positive GOM deposition and co‐segregation.^22^, ^23^ The role of other frequent variants including p.Pro167Ser, p.Arg1100His, and p.Gly1347Arg are still controversial so far.^24^, ^25^ Due to the controversial role of these mutations, we excluded them from the statistics in this study. However, the pathogenicity of these mutations needs to be further elucidated.

Although many valuable works have been done from the angle of CADASIL diagnosis, there is still no effective therapeutic strategy for CADASIL so far. Based on the genetic and clinical data analysis of CADASIL patients, fundamental research can be done from some new aspects. Firstly, pathogenicity of cysteine‐sparing mutations and mutations outside EGFR are ambiguous. For cysteine‐sparing mutations, interactions between amino acid residues may be a possible direction. Secondly, the mutation p.Arg544Cys is supposed to have a genotype–phenotype correlation in CADASIL patients. Since the mutation is located between two EGFRs, it may have a specific pathogenic mechanism that causes distinct clinical features. The role of this mutation also needs to be explored. Finally, gene therapy is a potential way for the disease. Specific therapies can be designed for population‐specific mutations. The mechanism and treatment of CADASIL needs to be further studied.

## 5 CONCLUSION

In summary, we identified two novel mutations (c.400T>C, p.Cys134Arg; c.1511G>A, p.Cys504Tyr) and 24 known cysteine‐affecting variants in the newly recruited CADASIL patients. We also analyzed and summarized the genetic spectrum of *NOTCH3* and clinical features of CADASIL patients in different populations. The results help to understand the clinical variability and genetic heterogeneity of CADASIL.

## CONFLICT OF INTEREST

The authors declare no conflicts of interest.

## Supporting information


Table S1

Table S2

## Data Availability

The data that support the findings of this study are available from the corresponding author upon reasonable request.
