# Supplementary material for: Genetic spectrum of NOTCH3 and clinical phenotype of CADASIL patients in different populations
Source: CNS Neurosci Ther. 2022 Jul 13;28(11):1779–89. doi: 10.1111/cns.13917 (PMC9532899; doi:10.1111/cns.13917)
Supplement: Supplementary file 1 — Table S1 [file CNS-28-1779-s002.docx]

| **Reference** | **Country/Year** | **Total pedigrees** | **Pedigrees with cysteine-involved mutations** |
| --- | --- | --- | --- |
| This study | China | 39 | 39 |
| ^1^ | China/2011 | 33 | 31 |
| ^2^ | China/2015 | 11 | 10 |
| ^3^ | China/2017 | 168 | 168 |
| ^4^ | China/2019 | 11 | 8 |
| ^5^ | China/2020 | 16 | 16 |
| ^6^ | China/2021 | 38 | 28 |
| ^7^ | China/2021 | 46 | 38 |
| ^8^ | China (Taiwan)/2015 | 95 | 95 |
| ^9^ | Japan/2015 | 62 | 54 |
| ^10^ | Japan/2017 | 9 | 7 |
| ^11^ | Japan/2020 | 179 | 165 |
| ^12^ | Korea/2006 | 9 | 5 |
| ^13^ | Korea/2014 | 29 | 19 |
| ^14^ | Korea/2019 | 32 | 23 |
| ^15^ | Korea/2020 | 70 | 47 |
| ^16^ | Korea/2021 | 157 | 102 |
| ^17^ | Europe/USA/North Africa/1997 | 45 | 45 |
| ^18^ | Dutch/2020 | 57 | 57 |
| ^19^ | Germany/2005 | 120 | 120 |
| ^20^ | Italy/2005 | 28 | 28 |
| ^21^ | Italy//2012 | 53 | 53 |
| ^22^ | Italy/2015 | 150 | 150 |
| ^23^ | Portugal/2021 | 23 | 17 |
| ^24^ | Turkey/2021 | 12 | 12 |
| ^25^ | UK/2004 | 64 | 64 |

**TABLE S1** Works included for genetic spectrum mapping

1. Wang Z, Yuan Y, Zhang W, et al. NOTCH3 mutations and clinical features in 33 mainland Chinese families with CADASIL. *J Neurol Neurosurg Psychiatry*. 2011;82(5):534-539.

2. Yin X, Wu D, Wan J, et al. Cerebral autosomal dominant arteriopathy with subcortical infarcts and leukoencephalopathy: Phenotypic and mutational spectrum in patients from mainland China. *Int J Neurosci*. 2015;125(8):585-592.

3. Chen S, Ni W, Yin XZ, et al. Clinical features and mutation spectrum in Chinese patients with CADASIL: A multicenter retrospective study. *CNS Neurosci Ther*. 2017;23(9):707-716.

4. Qin W, Ren Z, Xia M, et al. Clinical Features of 4 Novel NOTCH3 Mutations of Cerebral Autosomal Dominant Arteriopathy with Subcortical Infarcts and Leukoencephalopathy in China. *Med Sci Monit Basic Res*. 2019;25:199-209.

5. Wang Q, Huang Y, Xu QH, Han J, Yang MJ, Fu XJ. Analysis of clinical manifestations, MRI features and NOTCH3 gene mutation screening in CADASIL patients. *J Jinan Univ(Nat Sci Med Ed)* 2020;41(1):92-98.

6. Hu Y, Sun Q, Zhou Y, et al. NOTCH3 Variants and Genotype-Phenotype Features in Chinese CADASIL Patients. *Front Genet*. 2021;12:705284.

7. Zhang C, Li S, Li W, Niu S, Wang X, Zhang Z. Genotypic and Phenotypic Characteristics of Cerebral Autosomal Dominant Arteriopathy with Subcortical Infarcts and Leukoencephalopathy from China. *Eur Neurol*. 2021;84(4):237-245.

8. Liao YC, Hsiao CT, Fuh JL, et al. Characterization of CADASIL among the Han Chinese in Taiwan: Distinct Genotypic and Phenotypic Profiles. *PloS One*. 2015;10(8):e0136501

9. Ueda A, Ueda M, Nagatoshi A, et al. Genotypic and phenotypic spectrum of CADASIL in Japan: the experience at a referral center in Kumamoto University from 1997 to 2014. *J Neurol.* 2015;262(8):1828-1836.

10. Matsushima T, Conedera S, Tanaka R, et al. Genotype-phenotype correlations of cysteine replacement in CADASIL. *Neurobiol Aging*. 2017;50:169.e7-169.e14.

11. Mukai M, Mizuta I, Watanabe-Hosomi A, et al. Genotype-phenotype correlations and effect of mutation location in Japanese CADASIL patients. *J Hum Genet*. 2020;65(8):637-646.

12. Kim Y, Choi EJ, Choi CG, et al. Characteristics of CADASIL in Korea: a novel cysteine-sparing Notch3 mutation. *Neurology*. 2006;66(10):1511-1516.

13. Kim YE, Yoon CW, Seo SW, et al. Spectrum of NOTCH3 mutations in Korean patients with clinically suspicious cerebral autosomal dominant arteriopathy with subcortical infarcts and leukoencephalopathy. *Neurobiol Aging*. 2014;35(3):726.e1-726.e7266.

14. Kim Y, Lee SH. Novel Characteristics of Race-Specific Genetic Functions in Korean CADASIL. *Medicina (Kaunas)*. 2019;55(9):521.

15. Kim H, Lim YM, Lee EJ, Oh YJ, Kim KK. Clinical and imaging features of patients with cerebral autosomal dominant arteriopathy with subcortical infarcts and leukoencephalopathy and cysteine-sparing NOTCH3 mutations. *PloS One*. 2020;15(6):e0234797.

16. Min JY, Park SJ, Kang EJ, Hwang SY, Han SH. Mutation spectrum and genotype-phenotype correlations in 157 Korean CADASIL patients: a multicenter study. *Neurogenetics*. 2022;23(1):45-58.

17. Joutel A, Vahedi K, Corpechot C, et al. Strong clustering and stereotyped nature of Notch3 mutations in CADASIL patients. *Lancet*. 1997;350(9090):1511-1515.

18. Razvi SS, Davidson R, Bone I, Muir KW. Diagnostic strategies in CADASIL. *Neurology*. 2003;60(12):2019-2020.

19. Peters N, Opherk C, Bergmann T, Castro M, Herzog J, Dichgans M. Spectrum of mutations in biopsy-proven CADASIL: implications for diagnostic strategies. *Arch Neurol*. 2005;62(7):1091-1094.

20. Dotti MT, Federico A, Mazzei R, et al. The spectrum of Notch3 mutations in 28 Italian CADASIL families. *J Neurol Neurosurg Psychiatry*. 2005;76(5):736-738.

21. Testi S, Malerba G, Ferrarini M, et al. Mutational and haplotype map of NOTCH3 in a cohort of Italian patients with cerebral autosomal dominant arteriopathy with subcortical infarcts and leukoencephalopathy (CADASIL). *J Neurol Sci*. 2012;319(1-2):37-41.

22. Bianchi S, Zicari E, Carluccio A, et al. CADASIL in central Italy: a retrospective clinical and genetic study in 229 patients. *J Neurol*. Jan 2015;262(1):134-141.

23. Almeida MR, Elias I, Fernandes C, Machado R, Galego O, Santo G. NOTCH3 mutations in a cohort of Portuguese patients within CADASIL spectrum phenotype. *Neurogenetics*. 2022;23(1):1-9.

24. Rustemoglu BS, Samanci B, Tepgec F, et al. Clinical and Molecular Genetic Findings of Cerebral Arteriopathy with Subcortical Infarcts and Leukoencephalopathy. *Turk J Neurol*. 2021;27(3):240-247.

25. Singhal S, Bevan S, Barrick T, Rich P, Markus HS. The influence of genetic and cardiovascular risk factors on the CADASIL phenotype. *Brain*. 2004;127(Pt 9):2031-2038.
