# Supplementary material for: Genetic spectrum of NOTCH3 and clinical phenotype of CADASIL patients in different populations
Source: CNS Neurosci Ther. 2022 Jul 13;28(11):1779–89. doi: 10.1111/cns.13917 (PMC9532899; doi:10.1111/cns.13917)
Supplement: Supplementary file 2 — Table S2 [file CNS-28-1779-s001.docx]

**TABLE S2** Clinical manifestations of CADASIL patients from previous works

| **Reference** | **Country** | **Patients** | **Gender** | **Age at onset** | **Migraine** | **TIA/Ischemic stroke** | **Cognitive impairment** | **Psychiatric disturbance** | **External capsule** | **Temporal pole** |
| --- | --- | --- | --- | --- | --- | --- | --- | --- | --- | --- |
| This study | China | 39 | 19/39(48.72%) | 49.77±3.60 | 6/39(15.38%) | 29/39(74.36%) | 27/39(69.23%) | 10/39(25.64%) | 14/17(82.35%) | 12/17(70.59%) |
| ^1^ | China | 40 | 16/40(40%) | 40.95±2.42 | 3/40(7.5%) | 35/40(87.5%) | 16/40(40%) | 16/40(40%) |  | 16/40(40%) |
| ^2^ | China | 18 | 7/18(38.89%) | 38.67±4.02 | 5/18(27.78%) | 15/18(83.33%) | 7/18(38.89%) | 6/18(33.33%) | 14/18(77.78%) | 13/18(72.22%) |
| ^3^ | China | 216 | 97/216(44.91%) | 45±9 | 29/169(17.16%) | 115/169(68.05%) | 97/169(57.4%) | 56/169(33.14%) | 105/123(85.37%) | 94/123(76.42%) |
| ^4^ | China | 8 | 5/8(62.5%) |  | 2/8(25%) | 7/8(87.5%) | 2/8(25%) | 2/8(25%) | 4/8(50%) | 8/8(100%) |
| ^5^ | China | 15 | 9/15(60%) | 46.07±5.87 | 5/15(33.33%) | 9/15(60%) | 8/15(53.33%) | 4/15(26.67%) | 12/15(80%) | 7/15(46.67%) |
| ^6^ | China | 28 | 16/28(57.14%) | 44.96±8.82 | 7/28(25%) | 23/28(82.14%) | 13/28(46.43%) | 7/28(25%) | 24/28(85.71%) | 14/28(50%) |
| ^7^ | China (Taiwan) | 112 | 62/112(55.36%) | 54.1±12.5 | 3/112(2.68%) | 86/112(76.79%) | 46/112(41.07%) | 17/112(15.18%) | 82/96(85.42%) | 43/96(44.79%) |
| ^8^ | Japan | 44 | 24/44(54.55%) | 44.2±12 | 14/44(31.82%) | 30/44(68.18%) | 12/44(27.27%) | 9/44(20.45%) | 33/44(75%) | 34/44(77.27%) |
| ^9^ | Japan | 8 | 3/8(37.5%) | 53.8±15.5 | 1/8(12.5%) | 5/8(62.5%) | 5/8(62.5%) | 4/8(50%) | 8/8(100%) | 7/8(87.5%) |
| ^10^ | Japan | 165 | 82/165(49.7%) | 49.7 | 57/159(35.85%) | 128/165(77.58%) | 87/161(54.04%) | 33/159(20.75%) | 107/148(72.3%) | 143/163(87.73%) |
| ^11^ | Korea | 7 | 4/7(57.14%) | 50.29±10.95 | 1/7(14.29%) | 7/7(100%) | 3/7(42.86%) | 3/7(42.86%) |  |  |
| ^12^ | Korea | 19 | 7/19(36.84%) | 53.16±4.42 | 4/19(21.05%) | 11/19(57.89%) | 9/19(47.37%) | 1/19(5.26%) | 17/19(89.47%) | 11/19(57.89%) |
| ^13^ | Korea | 25 | 9/25(36%) | 52.6±3.82 | 8/25(32%) | 10/25(40%) |  |  | 16/24(66.67%) | 15/24(62.5%) |
| ^14^ | Korea | 55 | 28/55(50.91%) | 52.15±3.39 |  | 34/55(61.82%) | 11/55(20%) | 15/55(27.27%) | 35/55(63.64%) | 37/55(67.27%) |
| ^15^ | Korea | 102 | 33/102(32.35%) | 52.14 | 20/102(19.61%) | 62/102(60.78%) | 54/102(52.94%) | 18/102(17.65%) | 64/85(75.29%) | 52/85(61.18%) |
| ^16^ | Italy | 229 | 117/229(51.09%) | 48.5±17.1 | 95/229(41.48%) | 136/229(59.39%) | 88/229(38.43%) | 111/229(48.47%) |  |  |
| ^17^ | Turkey | 16 | 10/16(62.5%) | 43±6.65 | 1/16(6.25%) | 10/16(62.5%) | 8/16(50%) | 6/16(37.5%) |  | 11/14(78.57%) |
| ^18^ | Germany/Austria | 102 | 42/102(41.18%) | 37.0±13.5 | 39/102(38.24%) | 72/102(70.59%) | 49/102(48.04%) | 31/102(30.39%) |  |  |
| ^19^ | UK | 200 | 86/200(43%) | 33.6±14.1 | 150/200(75%) | 102/200(51%) | 34/200(17%) | 75/200(37.5%) |  |  |
| ^20^ | UK | 48 | 25/48(52.08%) | 35.9±14.6 | 31/48(64.58%) | 33/48(68.75%) |  |  | 43/46(93.48%) | 41/46(89.13%) |
| ^21^ | UK/Italy | 125 | 56/125(44.8%) | 33.4±17.1 | 84/125(67.2%) | 68/125(54.4%) | 46/125(36.8%) | 54/125(43.2%) | 96/125(76.8%) | 110/125(88%) |

1. Wang Z, Yuan Y, Zhang W, et al. NOTCH3 mutations and clinical features in 33 mainland Chinese families with CADASIL. *J Neurol Neurosurg Psychiatry*. 2011;82(5):534-539.

2. Yin X, Wu D, Wan J, et al. Cerebral autosomal dominant arteriopathy with subcortical infarcts and leukoencephalopathy: Phenotypic and mutational spectrum in patients from mainland China. *Int J Neurosci*. 2015;125(8):585-592.

3. Chen S, Ni W, Yin XZ, et al. Clinical features and mutation spectrum in Chinese patients with CADASIL: A multicenter retrospective study. *CNS Neurosci Ther*. 2017;23(9):707-716.

4. Qin W, Ren Z, Xia M, et al. Clinical Features of 4 Novel NOTCH3 Mutations of Cerebral Autosomal Dominant Arteriopathy with Subcortical Infarcts and Leukoencephalopathy in China. *Med Sci Monit Basic Res*. 2019;25:199-209.

5. Wang Q, Huang Y, Xu QH, Han J, Yang MJ, Fu XJ. Analysis of clinical manifestations, MRI features and NOTCH3 gene mutation screening in CADASIL patients. *J Jinan Univ(Nat Sci Med Ed)* 2020;41(1):92-98.

6. Hu Y, Sun Q, Zhou Y, et al. NOTCH3 Variants and Genotype-Phenotype Features in Chinese CADASIL Patients. *Front Genet*. 2021;12:705284.

7. Liao YC, Hsiao CT, Fuh JL, et al. Characterization of CADASIL among the Han Chinese in Taiwan: Distinct Genotypic and Phenotypic Profiles. *PloS One*. 2015;10(8):e0136501.

8. Ueda A, Ueda M, Nagatoshi A, et al. Genotypic and phenotypic spectrum of CADASIL in Japan: the experience at a referral center in Kumamoto University from 1997 to 2014. *J Neurol*. 2015;262(8):1828-1836.

9. Matsushima T, Conedera S, Tanaka R, et al. Genotype-phenotype correlations of cysteine replacement in CADASIL. *Neurobiol Aging*. 2017;50:169.e7-169.e14.

10. Mukai M, Mizuta I, Watanabe-Hosomi A, et al. Genotype-phenotype correlations and effect of mutation location in Japanese CADASIL patients. *J Hum Genet*. 2020;65(8):637-646.

11.Kim Y, Choi EJ, Choi CG, et al. Characteristics of CADASIL in Korea: a novel cysteine-sparing Notch3 mutation. *Neurology*. 2006;66(10):1511-1516.

12. Kim YE, Yoon CW, Seo SW, et al. Spectrum of NOTCH3 mutations in Korean patients with clinically suspicious cerebral autosomal dominant arteriopathy with subcortical infarcts and leukoencephalopathy. *Neurobiol Aging*. 2014;35(3):726.e1-726.e7266.

13. Kim Y, Lee SH. Novel Characteristics of Race-Specific Genetic Functions in Korean CADASIL. *Medicina (Kaunas)*. 2019;55(9):521.

14. Kim H, Lim YM, Lee EJ, Oh YJ, Kim KK. Clinical and imaging features of patients with cerebral autosomal dominant arteriopathy with subcortical infarcts and leukoencephalopathy and cysteine-sparing NOTCH3 mutations. *PloS One*. 2020;15(6):e0234797.

15. Min JY, Park SJ, Kang EJ, Hwang SY, Han SH. Mutation spectrum and genotype-phenotype correlations in 157 Korean CADASIL patients: a multicenter study. *Neurogenetics*. 2022;23(1):45-58.

16. Bianchi S, Zicari E, Carluccio A, et al. CADASIL in central Italy: a retrospective clinical and genetic study in 229 patients. *J Neurol*. 2015;262(1):134-141.

17. Rustemoglu BS, Samanci B, Tepgec F, et al. Clinical and Molecular Genetic Findings of Cerebral Arteriopathy with Subcortical Infarcts and Leukoencephalopathy. *Turk J Neurol*. 2021;27(3):240-247.

18. Dichgans M, Mayer M, Uttner I, et al. The phenotypic spectrum of CADASIL: clinical findings in 102 cases. *Ann Neurol*. 1998;44(5):731-739.

19. Adib-Samii P, Brice G, Martin RJ, Markus HS. Clinical spectrum of CADASIL and the effect of cardiovascular risk factors on phenotype: study in 200 consecutively recruited individuals. *Stroke*. 2010;41(4):630-634.

20. Markus HS, Martin RJ, Simpson MA, et al. Diagnostic strategies in CADASIL. *Neurology*. 2002;59(8):1134-1138.

21. Nannucci S, Rinnoci V, Pracucci G, et al. Location, number and factors associated with cerebral microbleeds in an Italian-British cohort of CADASIL patients. *PloS One*. 2018;13(1):e0190878.
